# Effect of a Theory-Informed, Six-Week Gamified Educational Intervention on Hydration Knowledge, Behavior, and Status in School Children: A Randomized Controlled Trial

**DOI:** 10.3390/nu18111753

**Published:** 2026-05-29

**Authors:** Sana Kacem, Khaled Trabelsi, Halil İbrahim Ceylan, Aïmen Khacharem, Achraf Ammar, Cain C. T. Clark, Kaïs El Abed, Haitham Jahrami, Raul Ioan Muntean, İsmail Dergaa, Nicola Luigi Bragazzi, Abdul Rashid Aziz

**Affiliations:** 1 High Institute of Sport and Physical Education of Sfax, University of Sfax, Sfax 3000, Tunisiaacammar@uni-mainz.de (A.A.); 2 Research Laboratory, Education, Motricity, Sport and Health (EM2S), LR15JS01, High Institute of Sport and Physical Education, University of Sfax, Sfax 3000, Tunisia; 3 Department of Movement Sciences and Sports Training, School of Sport Science, The University of Jordan, Amman 11942, Jordan; 4 Department of Physical Education of Sports Teaching, Faculty of Sports Sciences, Atatürk University, 25240 Erzurum, Türkiye; 5 College of Sport Sciences, Qatar University, Doha 2713, Qatar; 6 Department of Training and Movement Science, Institute of Sport Science, Johannes Gutenberg-University Mainz, 55122 Mainz, Germany; 7 Research Laboratory, Molecular Bases of Human Pathology, LR19ES13, Faculty of Medicine, University of Sfax, Sfax 3000, Tunisia; 8 Department of Nutrition and Food Technology, School of Agriculture, The University of Jordan, Amman 11942, Jordan; 9 School of Life and Health Sciences, Birmingham City University, Birmingham B15 3TN, UK; 10 Department of Psychiatry, College of Medicine and Medical Sciences, Arabian Gulf University, Manama 329, Bahrain; 11 Department of Physical Education and Sport, Faculty of Law and Social Sciences, University “1 Decembrie 1918” of Alba Iulia, 510009 Alba Iulia, Romania; 12 High Institute of Sport and Physical Education of Kef, University of Jendouba, Jendouba 7100, Tunisia; 13 Physical Activity Research Unit, Sport and Health (UR18JS01), National Observatory of Sports, Tunis 1003, Tunisia; 14 Department of Clinical Pharmacy, Saarland University, 66123 Saarbrücken, Germany; 15 Sport Science and Sports Medicine, Singapore Sport Institute, Singapore 397630, Singapore

**Keywords:** game-based intervention, behavior, health promotion, body hydration status, children, board game

## Abstract

**Aim:** This study assessed the effects of a six-week educational intervention using an adapted “Snakes and Ladders” board game on hydration knowledge, behavior, and status among Tunisian elementary school children during physical education (PE) lessons. **Method**: A randomized controlled trial involving 207 children was conducted, with participants assigned to either the educational group (EG, *n* = 99) or the control group (CG, *n* = 108). The EG participated in six weekly 30-min board game sessions, while the CG continued regular activities. Knowledge of hydration was assessed using a validated questionnaire. Hydration status was monitored indirectly by the percentage change in body mass from pre- to post-PE session. Perceived thirst was evaluated using a 9-point scale, and hydration behavior was evaluated based on water consumption during PE lessons. **Results**: Following the intervention, the EG demonstrated a significant improvement in overall hydration knowledge (ΔMean = +0.30 ± 0.11 vs. −0.05 ± 0.08 in CG; *p* < 0.001). Water intake during PE sessions increased progressively from week 2 (83.6 ± 127.2 mL) to week 6 (311.2 ± 204 mL) in the EG, whereas it remained unchanged in the CG (*p* < 0.001). Body mass loss after PE sessions decreased significantly in the EG (−0.03 kg) compared with the CG (−0.16 kg; *p* < 0.001), and perceived thirst before and after PE sessions was markedly lower (Cohen’s d = 0.75–1.32). **Conclusions**. The six-week board-game intervention appeared effective in increasing hydration knowledge, promoting healthier drinking behaviors during PE, and improving indirect indicators of hydration status. These findings provide preliminary evidence for the feasibility and educational value of a gamified, low-cost approach to hydration promotion in schools. Further research should examine long-term retention, include objective hydration biomarkers, and evaluate applicability across diverse school settings and environmental conditions.

## 1. Introduction

Maintaining proper hydration is crucial for health and well-being across all ages [1]. Indeed, adequate hydration is vital to various physiological processes, including temperature regulation, nutrient transport, and waste elimination [2]. For children, adequate hydration is crucial as it supports rapid growth, cognitive development, and physical performance [3]. Inadequate water consumption can lead to hypohydration, which negatively affects concentration, mood, and overall health [4]. The risks associated with hypohydration can increase when children engage in physical activities, such as routine training and physical education (PE) sessions, especially if they are already hypohydrated at the pre-exercise stage or do not maintain adequate water/fluid intake during these activities, particularly when exercising in hot and humid weather [5,6]. Therefore, ensuring that children maintain adequate hydration throughout the day, as well as before, during, and after physical activity, is crucial and requires the active involvement of parents, educators, and healthcare professionals [3].

Evidence indicates that inadequate hydration is common among children and adolescents; for instance, a study of 4134 children and adolescents aged 6 to 19 years in the U.S. found that over half (54.4%) were inadequately hydrated [4]. Furthermore, a systematic review examining water consumption across 19 countries found that approximately 60% of children aged 4–13 years did not meet the recommended daily water/fluid intake guidelines, indicating widespread hydration insufficiency [7]. Predominant factors contributing to inadequate hydration in children include limited access to water, a preference for sugary beverages, and a lack of awareness about the importance of drinking water. Recent reviews and school-based intervention studies have emphasized that inadequate water intake in children is a public health concern and that schools represent a key setting for promoting healthier hydration behaviors [8,9,10].

Studies conducted in sport and school contexts further highlight the magnitude of the problem. Indeed, Briefel et al. [1] carried out a study at a summer sports camp involving 13–14-year-old volleyball and basketball athletes and found that over 90% of participants arrived in a state of hypohydration. However, following a brief intervention, comprising a hydration lecture, a urine color chart, greater access to water, and body weight measurements before and after practice, their hydration status improved significantly within just two days. This improvement, achieved through volitional water intake, also led to enhanced endurance performance [1]. In another study by Decher et al. [11], hydration status and knowledge of 67 active young athletes were monitored during a 4-day summer sports camp. Throughout the camp, approximately 55% of the athletes experienced significant hypohydration levels (i.e., urine specific gravity was ≥1.025) [11]. However, the athletes demonstrated awareness of their hydration practices through questionnaire results, indicating that they recognized when they were hydrating well or poorly; however, they struggled to apply this knowledge effectively and failed to implement effective hydration strategies [11].

In a school setting, Michels et al. [12] reported that Belgian schoolchildren exhibited a concerning level of hydration behavior, with 76% being hypohydrated at the start of the day and 45% showing a decline in hydration levels throughout the school day. These findings suggest that many children may already begin their PE sessions in a hypohydrated state, highlighting the need for interventions to promote better hydration practices throughout the day; this is particularly important during PE classes, where the risk of hypohydration is elevated, and its health and injury risk consequences may be more significant [3].

Previous studies on interventions designed to improve water consumption among children can be categorized into three main types: educational health or nutritional programs (e.g., Martín-Payo et al. [13]), environmental policy changes aimed at encouraging water consumption (e.g., Kenney et al. [4]), and nutrition policies, such as banning sugary drinks at school events (e.g., Irwin et al. [14]). For instance, Martín-Payo et al. [13] conducted a feasibility study to assess the acceptability and effectiveness of an educational intervention that utilized web apps and posters, with the primary objective of improving water intake among adolescent soccer players. The intervention demonstrated high acceptability and led to positive changes in knowledge, attitudes, and behaviors related to water consumption [13]. However, these findings are specific to adolescents playing organized soccer and may not be generalizable to younger children or to other types of physical activity. Furthermore, the use of web apps and posters may be unsustainable, and such interventions may not have a long-term impact [15]. In contrast, Belogianni et al. [16] investigated the influence of an online educational program incorporating game-based learning on enhancing nutrition and physical activity outcomes among university students in the United Kingdom. The authors indicated that this type of intervention shows promising potential to improve both nutrition knowledge and physical activity behaviors among the university student population [16].

Similarly, Hassanzadeh-Rostami et al. [17] conducted a study that found game-based nutrition education significantly enhanced preschool children’s nutritional knowledge and understanding of key concepts. A systematic review and meta-analysis by Gauthier et al. [18], which examined the effectiveness of board games as a medium for health education, behavior change, and therapeutic interventions, concluded that board games could be implemented to successfully improve health knowledge, attitudes, and behaviors across various topics, including nutrition and physical activity.

To the best of our knowledge, no previous study has examined the effectiveness of any form of game-based/gamified intervention in improving children’s hydration knowledge, status, and practices, particularly in the context of PE classes. The intervention was conceptually informed by the Behavior Change Wheel (BCW) framework, specifically the COM-B model, which proposes that behavior change results from interactions among capability (C), opportunity (O), and motivation (M)–behavior (B) [19]. In the context of school PE, appropriate hydration practices depend not only on children’s knowledge and skills (capability) but also on the social and environmental conditions that enable drinking opportunities (opportunity) and children’s willingness to adopt healthy hydration habits (motivation). This framework, therefore, provided a structured basis for the design of the board game-based educational intervention.

This study, therefore, aims to evaluate the impact of a gamified intervention using a “Snakes and Ladders” board game on hydration knowledge, status, and practices among Tunisian schoolchildren before and during PE sessions.

The study hypothesized that the “Snakes and Ladders” intervention would significantly enhance the Tunisian children’s knowledge about hydration, improve their hydration practices, and positively affect their hydration status before and during PE classes. In addition, the findings of this study are expected to provide policymakers and school leaders with critical information to develop more effective strategies to promote adequate hydration among schoolchildren participating in PE sessions.

## 2. Materials and Methods

### 2.1. Design

This was a randomized controlled trial consisting of a board game educational intervention designed to improve the hydration status, knowledge, and practices of Tunisian schoolchildren aged 6–7 years during a PE session. Based on the study design, including its randomized nature, number of groups, and number of (repeated) measurements, an a priori power analysis was conducted to determine the minimum sample size required to detect a small effect size (d ≥ 0.2) and achieve a power (1 − β) of 0.95. Accordingly, a total sample of 166 participants would be required (83 per group). To account for potential attrition, we aimed for a sample size of ~200, with an equitable split between groups. However, due to the cluster allocation design, in which children in whole classes were assigned to the same group, slight deviations in group size were observed.

### 2.2. Participants

**Figure 1:**
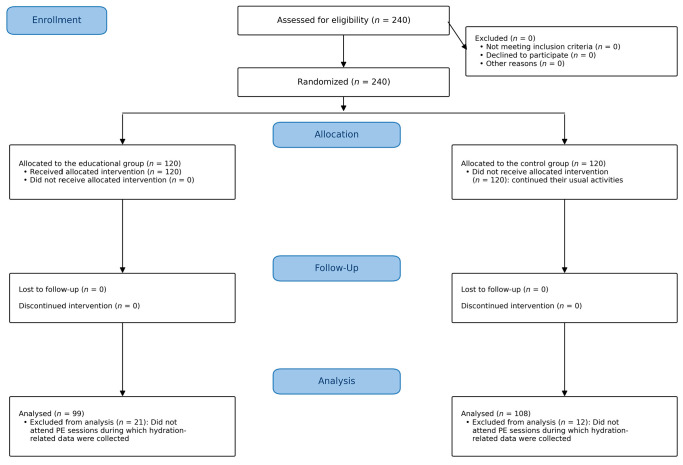
CONSORT Flow diagram of the study participants. The flow diagram illustrates enrollment, allocation, follow-up, and analysis of participants in a six-week board game-based educational intervention aimed at enhancing hydration knowledge, status, and practices among Tunisian schoolchildren. A total of 240 students were assessed for eligibility and randomly assigned to either the educational group (EG; *n* = 120) or the control group (CG; *n* = 120). During the intervention, 21 participants in the EG and 12 in the CG were excluded from analysis due to nonattendance at physical education sessions where hydration-related data were collected. Final analyses included 99 participants in the educational group and 108 in the control group.

Initially, 240 first-grade students, aged 6 to 7 years, from two public primary schools in Tunis, Tunisia, during the 2021–2022 academic year (February–March), volunteered to participate in this study. Written informed consent was obtained from parents prior to participation. In addition, verbal assent was obtained from all children using age-appropriate explanations of the study objectives and procedures, delivered in simple language with the assistance of classroom teachers. Children were informed that participation was voluntary and that they could stop at any time without any consequences. The inclusion criteria specified that children must have a normal body mass index (BMI) according to the Cole criteria [20]. Exclusion criteria included children who were exempted from PE classes due to previous injuries, allergies, or other medical conditions, as well as those absent from PE classes during the data collection period due to illness or injury. To avoid cross-contamination among participants, a cluster allocation design was used, grouping children from the same class together. The study was conducted in accordance with the Consolidated Standards of Reporting Trials (CONSORT) guidelines. Ethical approval for the study was granted by the South Persons Protection Committee (CPPSUD), with a favorable opinion issued under reference “CPP SUD N° 0372/2021”. A CONSORT flow diagram detailing participant enrollment, allocation, follow-up, and analysis is provided in Figure 1.

### 2.3. Intervention

The intervention was designed based on the BCW framework, specifically the COM-B model (Capability, Opportunity, Motivation–Behavior), to target key determinants of hydration-related behaviors in children. The educational content and board game mechanics were structured to enhance children’s knowledge and understanding of hydration (capability), provide supportive environmental cues during PE sessions (opportunity), and increase engagement and motivation through interactive and gamified learning (motivation).

### 2.4. Randomization and Blinding

This cluster-randomized controlled trial involved two primary schools, comprising eight first-grade classes (*n* = 240). Randomization was performed at the class level using a computer-generated sequence stratified by school to ensure balanced allocation. Because the intervention was educational, teachers and students were not blinded; however, the outcome assessors and data analysts remained blinded to the group allocation.

### 2.5. Measurement Instruments and Procedures

The present study uses four criteria to measure hydration. The first measure is the student’s knowledge of hydration. In both the educational group (EG) and control group (CG), each student’s hydration knowledge level was assessed before the educational interventions began and again before the start of week 6. Hydration knowledge was assessed using the Hydration Awareness Questionnaire [11], a previously validated instrument for children. The questionnaire was translated into Arabic by a bilingual sports scientist and independently reviewed by another bilingual researcher in sports sciences to ensure linguistic accuracy and conceptual equivalence between the original and translated versions. The questionnaire was administered in Arabic to ensure comprehension and was pilot-tested with 15 non-participating children; the pilot testing revealed no comprehension or administration difficulties. The Arabic adaptation showed satisfactory internal consistency in the present sample (Cronbach’s α = 0.81), although full psychometric validation remains warranted. The educational interventions were delivered weekly to the EG, while the CG followed their usual routine without intervention. The hydration knowledge questionnaire includes 22 Yes/No questions divided into four specific themes (T1, T2, T3, T4) (See Appendix A Table A1). The first one concerned a general knowledge of hydration (e.g., “water regulates the body’s temperature”, “adequate hydration should result in urine with a dark yellow hue”).

The remaining three themes are related to hydration practice (e.g., “consuming juice may be beneficial before physical activity”, “maintaining proper hydration is crucial during physical education activities”, “it is essential to drink a substantial amount of fluid immediately after engaging in physical activity”).

The second criterion is the child’s acute fluid balance as an indirect indicator of hydration status, measured as the percentage change in body weight before and after their PE lesson. The body mass (or weight) of each student was recorded using a digital scale (Aprilla, model ABS-1081; BMVA, Istanbul, Turkey) with a sensitivity of 0.1 kg before and after the PE session during the six-week intervention. Percentage change in body mass was determined using the equation described by Gibson et al. [1]:

Percentage body mass loss (%) = (body mass post-session – body mass pre-session)/body mass pre-session × 100.

Third, perceived thirst was measured before and after each PE session using a nine-point (1–9) Likert scale, where 1 indicated ‘not thirsty at all’ and 9 indicated ‘very, very thirsty’ [21]. Participants were asked, ‘How thirsty are you right now?’ when shown the scale, and they provided a numerical answer based on their perceived feeling of thirst. To support comprehension among the 6–8-year-old participants, the scale was administered verbally by a teacher who explained each numerical anchor using age-appropriate language.

Lastly, we quantified the amount of water each child consumed during the PE session by measuring the water in their individually marked bottles before and after the lesson.

All measurements were conducted weekly for six weeks, both before and after each PE session. PE teachers involved in the study followed standardized lesson plans for both the EG and the CG, ensuring consistency across 60-min PE sessions each week. These plans detailed the types of activities to be carried out, such as motor coordination exercises, as well as the overall structure of the lessons.

### 2.6. The Educational Board Game Intervention

The intervention consisted of six educational sessions based on the “Snakes and Ladders” board game. Each session lasted 30 min and was conducted once a week, immediately before the children’s PE class, over a six-week period. Participants in the CG received no intervention and continued their normal class activities. The educational board game was designed to align with the three components of the COM-B model [19]. Psychological capability was targeted through the nutritional messages embedded within the board squares and teacher-led reinforcement of these messages during gameplay. Social opportunity was supported through the structured group-based format, with the game played in small groups immediately before each PE session, normalizing hydration behaviors among peers. Reflective and automatic motivation were addressed through the reward–penalty mechanism of the game, where ladders symbolized the benefits of timely fluid intake and snakes represented the consequences of insufficient hydration, combined with the inherently gamified format, which leveraged children’s intrinsic motivation for play.

**Figure 2:**
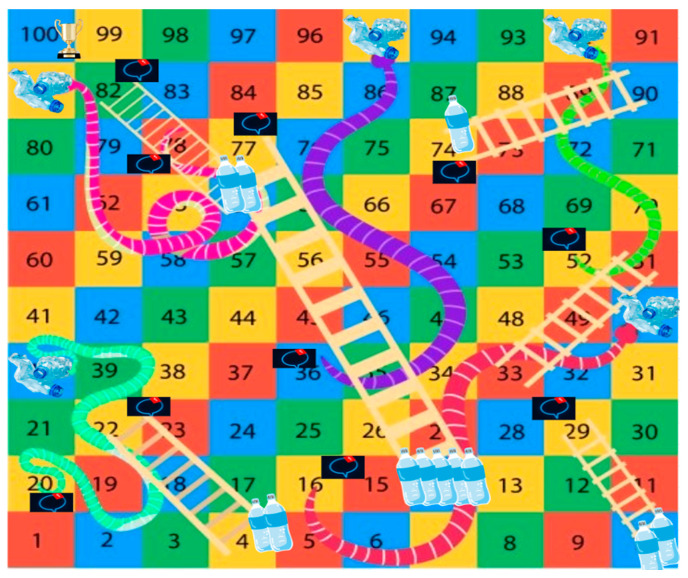
The board-based “Snakes and Ladders” game design.

The board game included 100 squares and was designed for up to four players per board (Appendix B, Table A2). Each child rolls a die to advance across the board to reach square #100 to win. Along the way, players encounter obstacles, such as snakes that send them backward and ladders that allow them to move forward more quickly. In this adapted version of the game, the focus is on promoting healthy hydration habits, with nutritional messages incorporated (Figure 2). For instance, when a player lands on a snake square, they experience a setback, symbolized by missing the opportunity to drink water, reinforcing the consequences of insufficient hydration. Conversely, landing on a square with a ladder rewards the player by allowing them to climb and receive a water bottle for fluid intake. Additionally, as they play, the children encounter and read positive messages about hydration, with close teacher support that reinforces these messages and lessons learned, making the board game engaging.

Various themes related to hydration knowledge and practices are incorporated into the game. Therefore, each week of the intervention period focuses on specific nutritional goals, which are integrated into the game mechanics (Table 1).

Ten minutes were designated before the start of the intervention to communicate the game’s guidelines and instructions to the study participants. 

### 2.7. Data Analysis

Descriptive statistics were calculated for all variables and are presented as means ± standard deviations (SDs) or frequencies and percentages, as appropriate. Data normality was assessed using the Kolmogorov–Smirnov test, as the sample size exceeded 50 participants [22]. Because the data were not normally distributed, non-parametric tests were used. Accordingly, between-group comparisons were performed using the Mann–Whitney U test, whereas within-group comparisons were performed using the Wilcoxon signed-rank test. Changes across repeated weekly measurements were analyzed using the Friedman test. When required, post hoc pairwise comparisons were adjusted using the Bonferroni correction.

Effect size (Cohen’s d) was calculated to quantify the magnitude of body mass changes, which were interpreted as small (0.2 ≤ d ≤ 0.5), moderate (0.5 ≤ d ≤ 0.8), or large (d > 0.8) [23]. Statistical significance was set at α < 0.01, and all analyses were conducted using IBM SPSS Statistics version 24.0. Moreover, Cohen’s d was reported as a descriptive standardized effect-size index to aid comparability with previous intervention studies. Because inferential testing used non-parametric procedures, d should be interpreted as an approximate magnitude indicator rather than a model-based estimate. Finally, although the study used class-level cluster allocation, cluster-adjusted analyses were not performed because only eight clusters were available; therefore, participant-level non-parametric analyses were retained.

## 3. Results

### 3.1. Description of the Sample and the Environmental Conditions

Of the 240 players initially selected, data from 33 participants (13.75%; *n* = 21 in the EG and *n* = 12 in the CG) were excluded due to absence during the PE sessions when water intake, body mass, and thirst perception-related data were collected; therefore, analyses were conducted using available cases. Consequently, a total of 207 children (mean age = 6.4 years, SD = 0.48) completed the study (*n* = 99 in EG and *n* = 108 in CG).

Baseline characteristics were comparable between the educational and control groups, with no significant differences in age, sex distribution, hydration knowledge, water intake during PE, body mass change, or thirst perception (Table 2). Environmental conditions were comparable for both groups throughout all weeks (Table 3).

### 3.2. Hydration Knowledge

The mean difference between the CG and EG for the questionnaire themes is presented in Table 4. After the six-week intervention, the delta in correct responses indicated a significant increase in the EG, while it remained unchanged in the CG. All themes covered in the questionnaire demonstrated significant positive changes in the EG, whereas the CG showed no change.

### 3.3. Hydration Practice

**Figure 3:**
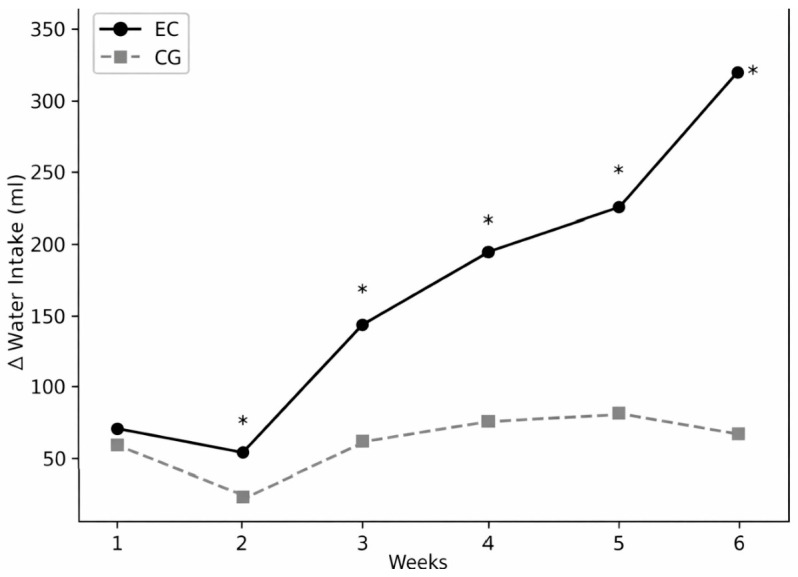
Changes in water intake across the 6 weeks for the educational and control groups. *: Significant differences compared to the control group (CG). Δ indicates the change between before and after PE sessions.

The water consumption during PE sessions in the EG and CG across the six weeks is presented in Table 5. No significant difference in water intake was observed between the groups during the first week. Still, from week 2 onward, water intake during PE sessions was significantly greater in the EG compared to the CG. In the EG, water intake increased significantly from week 2 to week 6 compared to week 1 (Figure 3). There was no significant difference in water intake for the CG across all PE sessions during the six weeks.

### 3.4. Indirect Indicators of Hydration Status

The change in body mass and thirst perception in the EG and CG over the six weeks is presented in Table 6 and Table 7, respectively. The results indicated a significantly greater decrease in body mass in the CG than in the EG across all weeks. Body mass loss during PE sessions in the EG was lower from week 2 through week 6 than in week 1.

For thirst perception, the EG showed a significant reduction compared to the CG, with effect sizes ranging from d = 0.75 (moderate) to d = 1.32 (large) over the weekly measurement period. Thirst perception in the EG was also lower during weeks 4, 5, and 6 compared to weeks 1, 2, and 3.

## 4. Discussion

To our knowledge, this study is the first to examine the effects of a board game-based educational intervention on hydration knowledge, status, and behavior before and during PE sessions in Tunisian children. The findings demonstrated that a once-weekly, game-based educational program over six weeks significantly improved hydration knowledge, behavior, and status among 6- to 7-year-old Tunisian elementary students, before and after PE sessions.

Interpreted through the COM-B framework, the findings suggest that the intervention influenced multiple determinants of hydration behaviors. Improvements in knowledge reflect enhanced psychological capability, while changes in hydration behaviors during PE may indicate increased opportunity and motivation to engage in appropriate drinking behaviors. This supports the usefulness of simple, theory-informed educational games for promoting healthy behaviors in school settings.

Each component of the COM-B framework appears to have contributed to this effect. The significant improvement in hydration knowledge in the EG indicates that the capability-targeting elements of the intervention, namely the weekly thematic messages and teacher reinforcement, successfully consolidated hydration-related knowledge in the participating children. The progressive increase in water intake during PE sessions from week 2 onward, together with the absence of any change in the CG, suggests that the structured group-based format created an effective social opportunity to drink, with the ladder squares providing repeated environmental cues. The reduction in body mass loss and perceived thirst across the intervention period is consistent with sustained motivation to act on the knowledge gained, supported by the reward–penalty structure of the game and the intrinsically rewarding playful format.

Beyond the theoretical framing, the design of the board game in the present study was inspired by a similar “Snakes and Ladders” game conducted with Iranian children aged 5–6 years [17], which aimed to improve general health through nutritional messages. In our study, the “Snakes and Ladders” game was specifically chosen to teach children about clean, healthy hydration practices. By making the learning process interactive, we aimed to better understand the importance of staying well-hydrated throughout the day, especially during PE sessions.

Overall, the game-based intervention effectively enhanced hydration knowledge in the EG, highlighting its positive impact on knowledge acquisition. While the specific knowledge gained may differ, our results align with those of Hassanzadeh-Rostami et al. [17].

Similarly, Hwang et al. [24] demonstrated that game-based education, using inquiry- and problem-solving-based approaches, could enhance student learning outcomes in nutrition knowledge, food attitudes, and habits compared with traditional methods.

Knowledge level is regarded as a significant predictor of water consumption habits and a key determinant of behavior change [14]. In our study, the EG demonstrated increased water intake during PE sessions from week 2 through week 6 compared with week 1, indicating positive hydration-related behavior. Furthermore, the EG consumed more water during physical activity than has been reported in previous studies [25,26].

Our results align with those of Kavouras et al. [27], who found that young athletes (adolescents) showed positive changes in hydration practices following a hydration lecture. Additionally, a systematic review by Racey et al. [28] indicated that interventions targeting adolescents in school settings can elicit positive changes in eating and drinking behaviors, including improved hydration practices. A meta-analysis by Vargas-Garcia et al. [29] quantified the effectiveness of health interventions aimed at reducing sugary drink consumption and increasing water intake, reporting a 67 mL/day increase in water consumption among children. However, Briefel et al. [1] reported that education alone did not alter hydration behaviors among adolescent athletes. Our study suggests that incorporating game-based interventions can significantly impact behavior change. This discrepancy may be attributed to the interactive and gamified nature of our intervention, which engages younger children more effectively than older children.

Another study investigated drinking behaviors and knowledge levels of primary school children aged 8 to 14 years in the UK, indicating that children with higher knowledge levels tended to consume fewer sugary drinks and more water [30]. This further supports the association between knowledge and behavior change. In a study by Martín-Payo et al. [13], an innovative educational intervention based on the BCW model, using a web app and posters, was well received by adolescent football players and led to improved hydration knowledge. However, the intervention did not significantly increase water consumption [13], aligning with other studies [1,31] that suggest that educational interventions can enhance knowledge without always engendering behavior change, particularly regarding hydration.

In contrast to the findings of Martín-Payo et al. [13], our results indicated improved hydration status in the EG, as evidenced by a decrease in delta body mass and changes in perceived thirst following PE sessions. Specifically, the percentage of body mass loss during PE sessions was less than 1%, suggesting that the children maintained adequate hydration throughout these activities. The decrease in thirst perception further supports the notion of adequate hydration during PE sessions. Notably, improvements in hydration status were observed as early as week 2 for delta body mass change and week 4 for change in thirst perception, indicating that the interactive board game-based intervention had an early impact. Furthermore, thirst perception before PE sessions was significantly lower in weeks 4, 5, and 6 than in the first three weeks, suggesting that the children may have been starting PE sessions more adequately hydrated. This improvement, which could positively impact both cognitive and physical performance during PE sessions, may be attributed to the knowledge gained through the board game-based educational intervention. It is important to note, however, that “hydration status” in the present analysis is inferred from indirect indicators (acute body mass change, perceived thirst, and recorded water intake) rather than from validated physiological biomarkers such as urine specific gravity or plasma osmolality, and that the between-group comparisons are unadjusted for class-level clustering; the present findings should therefore be read as supportive evidence of adequate fluid balance during PE rather than as direct measurement of true hydration status.

From a practical school health perspective, the magnitude of the observed effects is meaningful. In the EG, body mass loss during PE sessions remained below 1%, suggesting that children maintained adequate hydration throughout physical activity [32,33]. This level of hydration is generally considered sufficient to limit dehydration-related discomfort and may support better participation during PE lessons [34]. In addition, perceived thirst before and after PE sessions was significantly lower in the EG, with effect sizes ranging from moderate to large (Cohen’s d = 0.75–1.32), indicating a substantial improvement in hydration-related comfort. While improvements in hydration knowledge and drinking behavior are consistent with prior educational interventions, the present study is novel in demonstrating these effects through a low-cost, physical board game integrated into routine PE sessions among early primary school children.

Interestingly, Neumark-Sztainer et al. [35] reported that establishing healthy eating and drinking behaviors in early childhood can foster healthy habits later in life. Consequently, the current educational board game intervention may enable first-year primary school students to acquire hydration knowledge, which can serve as a foundation for essential behaviors during PE sessions in subsequent years [35].

We acknowledge that the present findings stem from a six-week intervention conducted in a specific Tunisian school context and should be interpreted with appropriate caution. The trial’s short duration, the young age of participants (6–7 years), the cluster allocation by class, and the use of primarily behavioral and anthropometric measures (body mass change, self-reported thirst, and recorded water intake) limit the strength of causal claims and generalizability. Additionally, data were collected during a mild climatic period and within specific school routines and cultural practices that may influence hydration behaviors. For these reasons, we describe the results as promising preliminary evidence rather than definitive proof of sustained physiological benefit.

Nevertheless, the consistent improvements in knowledge, drinking behaviors during PE, and reduced body mass loss suggest the intervention warrants replication. Future studies should test this approach in other climatic conditions and school systems, include different age groups, monitor training load to ensure equivalent exercise stimulus, and incorporate objective biomarkers of hydration (e.g., urine specific gravity) as well as longer-term follow-up to evaluate durability and scalability.

From an implementation perspective, this intervention appears feasible for school settings. The board game is low-cost, requires minimal materials, and can be delivered within existing PE schedules through short weekly sessions led by teachers. With brief teacher orientation and basic administrative support (e.g., scheduling and ensuring access to drinking water), the intervention could be readily integrated into broader school health promotion curricula. This approach aligns with current understanding of children’s hydration as a public health priority [8] and with survey evidence that teachers are central mediators of children’s fluid behavior [9]. It is also supported by recent cluster-randomized trial findings showing that classroom-based water promotion can improve children’s beverage choices and weight outcomes [10].

### Limitations and Future Directions

While this study provides valuable insights into hydration knowledge, status, and behaviors, certain limitations should be acknowledged. An important methodological limitation is the cluster-randomized design, as statistical analyses did not explicitly account for potential within-class non-independence. This decision was driven by the limited number of clusters available (eight, four per arm), which restricts the reliability of multilevel modelling approaches. Although nonparametric tests were appropriate given the non-normal distribution of the data, they do not fully address clustering effects. Future studies with a larger number of clusters should apply cluster-adjusted or multilevel analytical methods to confirm and extend these findings.

In addition, the use of an Arabic version of the Hydration Awareness Questionnaire without a formal cross-cultural validation process represents another methodological limitation. Although the instrument demonstrated acceptable internal consistency in the present sample, future studies should conduct a comprehensive cross-cultural validation, for example, following the recommendations of Beaton et al. [36], to further strengthen measurement validity and precision.

Beyond this methodological consideration, future research should further optimize these interventions by incorporating feedback from the target audience, tailoring content to different age groups, and examining their long-term effects on hydration habits. Furthermore, the effectiveness of digitally delivered interventions should be evaluated; however, implementing and sustaining these interventions in low- and middle-income countries can pose significant challenges. Moreover, future studies should incorporate heart rate monitoring during PE lessons to confirm that both groups experience equivalent training loads.

Given that this study was conducted in a mild climate, it is crucial to evaluate the intervention’s effectiveness in hot, humid environments, as these conditions can significantly affect hydration status and behaviors [37]. Last, while challenging, future research should prioritize the use of more objective hydration measures (e.g., urine specific gravity, urine color) to strengthen the validity of the findings.

## 5. Conclusions

This study provides preliminary evidence that early childhood nutritional education delivered through interactive, game-based learning can enhance hydration knowledge, foster healthier drinking behaviors, and improve indirect indicators of hydration status among schoolchildren before and during PE sessions. The findings highlight the potential of gamified, board-game-based interventions as engaging, low-cost, and sustainable tools for health promotion in school settings. By integrating gamified learning strategies into the curriculum, educators can effectively foster long-term, positive habits that support both physical and cognitive well-being.

Future research should aim to optimize these interventions by tailoring them to diverse age groups and educational contexts, examining their long-term impact on daily hydration habits, and incorporating more objective physiological measures of hydration. Expanding this approach to different climates and cultural environments may also provide valuable insights into its broader applicability and effectiveness in promoting children’s overall health and well-being.

## Figures and Tables

**Table 1:** The objectives of each week of the “Snakes and Ladders” board game intervention sessions.

Week	Themes
1	The benefits of hydration
2	Recommended hydration sources
3	The importance of drinking water before and after PE
4	The signs and dangers of dehydration
5	Prevention of dehydration, before, during, and after PE
6	Determinants of beneficial hydration practices

PE: physical education.

**Table 2:** Baseline characteristics of the educational and control groups.

Variable	EG (*n* = 99)	CG (*n* = 108)
Age mean ± SD	6.34 ± 0.47	6.38 ± 0.49
Sex (%)	Girls: 54.54% Boys: 45.45%	Girls: 51.85% Boys: 48.15%
Hydration knowledge score mean ± SD	0.49 ± 0.03	0.47 ± 0.03
Hydration knowledge by theme (T1–T4) Mean ± SD	T1: 0.45 ± 0.19 T2: 0.36 ± 0.19 T3: 0.63 ± 0.17 T4: 0.50 ± 0.24	T1 0.44 ± 0.21 T2 0.33 ± 0.18 T3 0.61 ± 0.17 T4 0.49 ± 0.25
Water intake during PE (mL), week 1	47.40	66.12
Delta body mass change during PE, week 1	−0.27 ± 0.73	−0.78 ± 0.41
Thirst score pre-PE (A.U), week 1	6.47 ± 2.81	7.83 ± 1.81
Thirst score post-PE (A.U), week 1	6.01 ± 2.81	7.69 ± 1.61

A.U: arbitrary unit; PE: Physical Education; EG: Educational Group; CG: Control Group.

**Table 3:** Temperature and percentage humidity during the physical education sessions.

Week	EG Temperature (°C)	EG Humidity (%)	CG Temperature (°C)	CG Humidity (%)
1	17.50 (0.60)	51.50 (4.50)	17.00 (0.70)	58.00 (5.00)
2	17.00 (0.80)	55.00 (5.00)	17.80 (0.90)	63.90 (4.50)
3	17.30 (0.70)	63.80 (3.50)	17.40 (0.80)	65.10 (4.00)
4	16.30 (0.60)	64.50 (4.00)	17.50 (0.70)	63.50 (3.50)
5	18.00 (0.90)	53.60 (4.50)	18.30 (1.00)	53.20 (4.50)
6	20.00 (1.20)	65.50 (4.00)	21.00 (1.30)	56.00 (5.00)

Mean ± SD values are reported. EG: Educational Group; CG: Control Group.

**Table 4:** Delta-change intervention means for the questionnaire’s themes for the CG and EG.

Theme	EG: *n* = 99	CG: *n* = 108	*p*-Value
ΔT1	0.32 ± 0.18	−0.02 ± 0.19	0.001
ΔT2	0.43 ± 0.21	0.01 ± 0.11	0.001
ΔT3	0.18 ± 0.14	−0.10 ± 0.11	0.001
ΔT4	0.29 ± 0.24	−0.07 ± 0.16	0.001
Δ Mean	0.30 ± 0.11	−0.05 ± 0.08	0.001

Nonparametric tests (Wilcoxon’s signed-rank test); EG: educational group; CG: control group; T1: theme number one; T2: theme number two; T3: theme number three; T4: theme number four.

**Table 5:** Water intake during PE sessions (mL).

Week	Group	Mean ± SD	*p*-Value *	Cohen’s d
Week 1	EG	47.40 ± 95.47	0.517	0.15
	CG	66.12 ± 146.74		
Week 2	EG	83.55 ± 127.20 ^a^	<0.001	0.33
	CG	41.00 ± 121.40		
Week 3	EG	151.31 ± 197.70 ^a^	<0.001	0.44
	CG	70.75 ± 156.85		
Week 4	EG	217.60 ± 180.74 ^a^	<0.001	0.78
	CG	79.00 ± 169.18		
Week 5	EG	242.07 ± 170.41 ^a^	<0.001	0.75
	CG	89.50 ± 178.56		
Week 6	EG	311.15 ± 204.00 ^a^	<0.001	1.44
	CG	53.99 ± 141.00		

Mean ± SD values are reported. Between-group comparisons used the Mann–Whitney U test (data were non-normally distributed); within-group comparisons used the Wilcoxon signed-rank test. EG = educational group; CG = control group; SD = standard deviation; PE = physical education. ^a^ Significant within-group increase in water intake relative to week 1. * *p*-values are unadjusted for class-level clustering.

**Table 6:** Acute change in body mass during PE sessions (kg).

Week	Group	Median (IQR)	*p*-Value *	Cohen’s d
Week 1	EG	−0.07 (−0.11; −0.04)	<0.001	0.86
	CG	−0.20 (−0.25; −0.14)		
Week 2	EG	−0.09 (−0.14; −0.01) ^a^	<0.001	0.70
	CG	−0.15 (−0.20; −0.10)		
Week 3	EG	−0.09 (−0.17; 0)	<0.001	0.80
	CG	−0.18 (−0.22; −0.12)		
Week 4	EG	−0.09 (−0.18; 0)	<0.001	0.68
	CG	−0.19 (−0.22; −0.12)		
Week 5	EG	−0.12 (−0.21; 0)	<0.001	0.75
	CG	−0.20 (−0.24; −0.12) ^a^		
Week 6	EG	−0.03 (−0.13; 0) ^a^	<0.001	0.55
	CG	−0.16 (−0.25; −0.09)		

Median (IQR) values are reported. Between-group comparisons used the Mann–Whitney U test; within-group comparisons used the Wilcoxon signed-rank test. EG = educational group; CG = control group; IQR = interquartile range; PE = physical education. ^a^ Significant within-group difference relative to week 1. * *p*-values are unadjusted for class-level clustering.

**Table 7:** Perceived thirst before and after PE sessions and within-PE change in thirst on a 9-point scale.

Week	Group	Pre-PE Median (IQR)	Post-PE Median (IQR)	Δ Thirst Median (IQR)	*p*-Value *	Cohen’s d
Week 1	EG	6.00 (4.00; 9.00)	8.00 (3.00; 9.00)	2.00 (3.00; 9.00)	0.004	0.57
	CG	8.00 (6.00; 9.00)	8.00 (8.00; 9.00)	0.00 (6.00; 9.00)		
Week 2	EG	5.00 (1.00; 9.00)	7.00 (3.00; 8.00)	2.00 (1.00; 9.00)	<0.001	0.73
	CG	8.00 (7.00; 9.00)	8.00 (8.00; 9.00)	0.00 (7.00; 9.00)		
Week 3	EG	6.00 (3.00; 8.00)	5.00 (2.00; 9.00) ^a^	−1.00 (2.00; 9.00)	<0.001	1.01
	CG	7.00 (7.00; 9.00)	8.00 (8.00; 9.00)	1.00 (7.00; 9.00)		
Week 4	EG	2.00 (1.00; 9.00) ^b^	3.00 (1.00; 9.00) ^a^	1.00 (1.00; 9.00)	<0.001	1.08
	CG	8.00 (8.00; 9.00)	8.00 (8.00; 9.00)	0.00 (8.00; 9.00)		
Week 5	EG	2.00 (2.00; 8.00) ^b^	3.00 (1.00; 9.00) ^a^	1.00 (1.00; 9.00)	<0.001	0.85
	CG	8.00 (8.00; 9.00)	8.00 (7.00; 9.00)	0.00 (7.00; 9.00)		
Week 6	EG	1.00 (1.00; 8.00) ^b^	2.00 (1.00; 8.00) ^a^	1.00 (1.00; 8.00)	<0.001	1.32
	CG	7.00 (6.00; 9.00)	8.00 (8.00; 9.00)	1.00 (6.00; 9.00)		

Median (IQR) values are reported. Between-group comparisons used the Mann–Whitney U test on Δ thirst; within-group comparisons used the Wilcoxon signed-rank test. EG = educational group; CG = control group; IQR = interquartile range; PE = physical education. ^a^ Significant within-group reduction in post-PE thirst relative to week 1. ^b^ Significant within-group reduction in pre-PE thirst relative to weeks 1–3. * *p*-values are unadjusted for class-level clustering.

## Data Availability

Data associated with this paper can be produced on request from the corresponding author.

## Trial Registration

This study is registered with Pan African Clinical Trial Registry (PACTR) under the unique identifier (PACTR202510771129829) on 6 October 2025.

## Appendix A

**Table A1:** List of questions to assess the hydration knowledge of children.

Theme	YES/NO Questions
T1	Adequate hydration is maintaining the appropriate fluid levels in the body.
	Adequate hydration should result in urine with a dark yellow hue.
	Water regulates the body’s temperature.
	Drinking water when not thirsty is recommended.
	Maintaining adequate hydration is critical in a hot environment to meet the body’s increased fluid requirements.
T2	Maintaining proper hydration is crucial before participating in physical education and sports activities.
	Consuming juice may be beneficial before physical activity.
	Consuming fruit-based liquids is an optimal hydration strategy prior to physical activity.
	Consuming carbonated drinks (e.g., cola) can benefit individuals before physical exercise.
	Consuming carbonated water is optimal before exercise.
	Consuming water before physical activity is associated with increased body mass.
T3	Maintaining proper hydration is crucial during physical education activities.
	When engaging in physical activity, thirst indicates the body is losing water and requires replenishment.
	Adequate hydration is not necessary for optimal sports performance.
	Increased physical activity may necessitate greater hydration.
	Increased fluid intake is recommended when physical activity is more intense than usual.
	Consuming fruit-based liquids represents an optimal hydration strategy during physical activity.
	Consuming carbonated drinks (e.g., cola) can benefit individuals during physical exercise.
	Consuming carbonated water is optimal during exercise.
T4	It is essential to drink a substantial amount of fluid immediately after engaging in physical activity.
	It is important to rehydrate promptly after physical exertion.
	Drinking water is sufficient for maintaining proper hydration after exercise.
	Adequate hydration is required to replace the fluids lost through perspiration during physical activity.

T1: theme number one; T2: theme number two; T3: theme number three; T4: theme number four.

## Appendix B

**Table A2:** Educational board game intervention.

Component	Description
Board layout & structure	Adapted Snakes and Ladders board with 100 numbered squares, for 4 players. Players advance by rolling a die. Landing on a snake moves backward; the ladder moves forward. Objective: reach square 100.
Hydration adaptations	Snakes = setbacks from inadequate hydration (e.g., skipping water). Ladders = positive hydration behaviors (e.g., drinking water). Messages are embedded in squares and reinforced verbally.
Weekly educational themes	Six weekly sessions, each with a hydration objective. Messages are adapted weekly to match the theme.
Example messages	Week 1: “Drinking water helps your body stay active and strong.” Week 2: “Water is the best drink for your body.” Week 3: “Drinking water after PE helps your body recover.” Week 4: “Not drinking enough water can make you tired.” Week 5: “Drink water before, during, and after PE.” Week 6: “Remembering to drink water is a healthy habit.”
Teacher’s role	Supervised sessions, explained rules, facilitated turns, and reinforced messages. First 10 min dedicated to game rules and objectives.

PE: physical education.
